# *Miconia* sp. Increases mRNA Levels of PPAR Gamma and Inhibits Alpha Amylase and Alpha Glucosidase

**DOI:** 10.1155/2016/5123519

**Published:** 2016-07-13

**Authors:** David Mizael Ortíz-Martinez, Catalina Rivas-Morales, Myriam Angelica de la Garza-Ramos, Maria Julia Verde-Star, Maria Adriana Nuñez-Gonzalez, Catalina Leos-Rivas

**Affiliations:** ^1^Facultad de Ciencias Biologicas, Universidad Autonoma de Nuevo Leon, Pedro de Alba, s/n, Ciudad Universitaria, 66455 San Nicolas de los Garza, NL, Mexico; ^2^Facultad de Odontologia, Universidad Autonoma de Nuevo Leon, Eduardo Aguirre Pequeño y Silao, s/n, Mitras Centro, 64460 Monterrey, NL, Mexico

## Abstract

Diabetes mellitus is a public health problem worldwide. For this reason, ethanolic extract of* Miconia* sp. from Oaxaca, Mexico, was selected in search of an alternative against this disease. The effect of* Miconia* sp. on mRNA expression of PPAR*γ* on cell line 3T3-L1, its effect on alpha amylase and alpha glucosidase, lipid accumulation during adipogenesis, and cell viability on VERO cells were evaluated. The mRNA levels of PPAR*γ* increased on 1.393 ± 0.008 folds, lipid accumulation was increased by 29.55% with* Miconia* sp. extract and 34.57% with rosiglitazone, and *α*-amylase and *α*-glycosidase were inhibited with IC_50_ values from 28.23 ± 2.15 *μ*g/mL and 1.95 ± 0.15 *μ*g/mL, respectively; the IC_50_ on antiproliferative activity on VERO cells was 314.54 ± 45.40 *μ*g/mL. In case of *α*-amylase and *α*-glycosidase assays, IC_50_ (inhibitory concentration 50) refers to necessary extract amounts to inhibit 50% of enzymatic activity. On the other hand, on antiproliferative activity, IC_50_ (inhibitory concentration 50) refers to necessary extract amounts to inhibit 50% of cell proliferation. It was concluded that the compounds present in* Miconia* sp. ethanolic extract increase mRNA expression of PPAR*γ*, inhibit *α*-amylase and *α*-glucosidase, and increase lipid accumulation. It constitutes an alternative as adjuvant in diabetes mellitus treatment; therefore, we recommend continuing identifying the compounds responsible for its promising in vivo antidiabetic activity.

## 1. Introduction

Diabetes mellitus is a chronic metabolic disease considered a serious global public health problem. In 2010, approximately 285 million people suffered from this disease and this amount is expected to double up within the next 20 years [1]. Diabetes mellitus type 2 (DM2) is the most common form of diabetes. It is a complex metabolic alteration characterized by an insulin combination resistance (IR, low sensitivity of one or multiple tissues to insulin) and insulin secretion alteration [2].

The search for new drugs that act against peroxisome proliferator-activated receptor gamma (PPAR*γ*) is very important because ligands of these transcription factors exhibit multiple biological responses such as decreasing the IR and avoiding high levels of plasm glucose. It has been shown that the adipogenesis process is under the control of a complex cascade of transcriptional regulatory factors in which PPAR*γ* and other transcriptional factors of C/EBP family play a fundamental role [3, 4].

Enzymes *α*-amylase and *α*-glucosidase found in saliva and the brush border of the small intestine, respectively, act on hydrolysis oligosaccharides and disaccharides to produce easy absorption monosaccharides such as glucose. For the above mentioned, delaying absorption of glucose through inhibition of enzymatic hydrolysis of carbohydrates, carried out by alpha amylase and alpha glucosidase, could be another form of combating DM [4].

About 80% of the population worldwide use medicinal plants to treat various diseases. These constitute a major source of drugs; about 25% of prescribed drugs worldwide originate from plant species [5].

A species of the genre* Miconia* in Mexican traditional medicine is used in south Mexico as an alternative for diabetes mellitus treatment. However, such fact has not been scientifically confirmed yet.* Miconia* is a genus of about 1000 species distributed in tropical America and belongs to the Melastomataceae family, which has about 4300 species distributed in 166 genera. Very few studies on biological activity have been performed on this genre, but it has been shown that extracts and compounds isolated from species of the genus* Miconia* have antibiotic, antitumor, analgesic, and antimalarial activities. A phytochemical analysis of methanolic extract of* Miconia cabucu* reveals the presence of glycosylated flavonoids (quercetin, myricetin, and kaempferol all with different glycosides), a tannin, and rare bioflavonoid. The phytochemical research of* M. rubiginosa* extract led to the identification of several glycosylated forms such as quercetin, gallic acid, and epicatechin. Similarly, quercetin, myricetin, and catechin derivatives* M. stenostachya* were found [6].

## 2. Materials and Methods

### 2.1. Preparation of the Extract

*Miconia* sp. aerial part was collected in Oaxaca, Mexico. The plant was dried in shade at room temperature and extraction with ethanol was performed by the Soxhlet method (PYREX) for 48 h; solvent was removed under reduced pressure using a rotary evaporator (Yamato RE-200). Extract yield was determined and then stored at room temperature until it was used.

### 2.2. Cell Culture Preparation and Adipocyte Differentiation

Murine cell line 3T3-L1 preadipocytes were used. DMEM medium supplemented with 10% fetal bovine serum (FBS) was used for propagation; it was incubated at 37°C at 5% CO_2_ atmosphere, until reaching a confluence of 90%. The 100% confluency was used for adipocyte differentiation. It could be DMEM with 10% FBS, 1 *μ*M dexamethasone, 0.5 mM 3-isobutyl-1-methylxanthine, and 10 *μ*g/mL insulin (all brands from Santa Cruz Biotechnology). After 48 hours of incubation (day 2), the differentiation medium was removed and replaced every 48 hours until maturation of adipocytes (days 9–11), with maturation medium of adipocytes (DMEM with 10% FBS and insulin 10 *μ*g/mL called MM). The maturation was confirmed by microscope observation of the typical morphology of an adipocyte [7–10].

### 2.3. Expression of PPAR*γ* in Adipocytes

The study was performed in the cell line 3T3-L1 differentiated, following the methodology described above, using microplate 6-well culture with an inoculum of 100,000 cells per well in a final volume of 2 mL with the corresponding culture medium. At day 10, adipocyte differentiation was observed and replaced in the medium (MM). The following treatments were added: Group 1: DMEM with 0.1% ethanol (expression control group), Group 2: MM supplemented with 1 *μ*M of rosiglitazone (positive control), Group 3: MM supplemented with* Miconia* sp. extract (40 *μ*g/mL), and Group 4: MM only to observe the effect of the medium. After 24 hours of treatment application of the total RNA extraction, the retrotranscription (cDNA) and PCR were performed in real time to determine the effect on mRNA expression [8–10].

The total RNA isolation of treated adipocytes was performed with an extraction kit SV Total RNA Isolation System # Z3100, Promega (following the manufacturer's instructions) and stored at −80°C until use. Total RNA integrity, purity, and quantification analysis was made in NanoDrop 8000 Spectrophotometer with Thermo Scientific and an electrophoresis gel (1.5% agarose).

CDNA synthesis was done with the ImProm-II*™* Reverse Transcription System kit Promega # A3800 with 500 ng of RNA isolated. The cDNA was stored at −20°C, until use.

Analysis of mRNA expression PPAR*γ* was performed by qRT-PCR using LightCycler 480 II Roche equipment, and 50 ng of cDNA, 0.5 *μ*M of the primers, 12.5 *μ*L of Maxima SYBR Green qPCR Master Mix (2x) # K0251 Thermo Scientific, and the proper amount of nuclease-free water to have a final volume of 25 *μ*L. The 36B4 was used [Thomson] as reference gene. The sequence of the primers used is PPAR*γ* forward 5′-CTGGCCTCCCTGATGAATAAAG-3′, reverse 5′-AGGCTCCATAAAGTCACCAAAG-3′, 36B4 forward 5′-ACTGGTCTAGGACCCGAGAAG-3′, and reverse 5′-TCAATGGTGCCTCTGGAGATT-3′. The relative mRNA expression was calculated based on the 2^−ΔΔCt^ method.

### 2.4. Lipid Accumulation in Adipocytes

Lipid accumulation was evaluated on the cell line 3T3-L1 using Oil Red O, with modifications to the method described by other authors. The method described above was used for cell differentiation. Microculture plates were used having 24 wells with an inoculum of 30,000 cells per well leading to a final volume of 500 *μ*L with culture medium. At 48 h after confluence (day 0) the differentiation medium (Dm) was applied to stimulate adipogenesis, supplemented with the substances to be evaluated: treatment 1 [Dm (control reference)], treatment 2 [Dm supplemented with 40 *μ*g/mL extract* Miconia* sp.], and treatment 3 [Dm supplemented with 1 *μ*M rosiglitazone (positive control)]. Subsequently, the microplate was incubated for 48 h with the established conditions. After that, Dm was replaced by MM (day 2) and incubated for 8 d. The culture medium was removed, and formalin 10% was used for 1 h to fix monolayer cells. After monolayer cells had been washed twice with distilled water, the dye Oil Red O (0.5% in 60% isopropanol) was applied for 15 min at room temperature. The cells were washed three times with distilled water; the dye inside the cells was removed with 100% isopropanol and read the optical density at 540 nm [9–12].

### 2.5. Inhibitory Activity of *α*-Amylase

The enzyme inhibition was evaluated by the method of dinitrosalicylic acid 3,5-(DNS) with some modifications. The* Miconia* sp. extract was applied at different concentrations (25, 50, 75, 100, and 125 *μ*g/mL) as a vehicle using phosphate buffer with 20 mM sodium 6.7 mM sodium chloride, at pH 6.9. *α*-Amylase enzyme (Sigma-Aldrich # A9857-250KU) was used at 0.5 U/mL concentration with the same buffer as a vehicle. Starch 0.5% in phosphate buffer was used as a substrate. Acarbose at different concentrations (312.5, 625, 1250, 2500, and 5000 *μ*g/mL) was used as a positive control. Additionally, DNS solution was used at 96 mM. In 1.5 mL conical tubes, 50 *μ*L of substances was placed to be evaluated: treatment 1:* Miconia* sp. extracts, treatment 2: buffer phosphate with 2% ethanol (negative control), and treatment 3: acarbose (positive control). Briefly, to the above treatments 50 *μ*L of the *α*-amylase was added and incubated at 37°C for 10 min. After that, to initiate the reaction, 50 *μ*L of the substrate was added immediately and incubated at 37°C for 15 min. Then, the reaction was stopped by adding 50 *μ*L of DNS and heated in a water bath at 95°C for 5 min; the tubes were left to cool at room temperature, and the optical density was measured at 540 nm. The absorbance rates were used to calculate the percent inhibition of each treatment using the following equation:

(1) $$\begin{array}{c} \%\;\;\text{Inhibition}=\frac{\left(\text{Abs control}-\text{Abs treatment}\right)}{\text{Abs control}}\times 100. \end{array}$$

In this equation, “Abs treatment” is the product's light absorption of the enzyme-substrate reaction in the presence of the* Miconia* sp. or negative control as appropriate. “Abs control” is the reaction product's light absorption of enzyme-substrate in the presence of phosphate buffer as a treatment. The percentage inhibition using the statistical package (SPSS v.20) allows us to calculate the inhibitory concentration: 50 (IC_50_) is the amount required to inhibit 50% of the enzyme (Sigma-Aldrich # A8980-1G) [5, 13–15].

### 2.6. *α*-Glucosidase Inhibitory Activity

Inhibition of the enzyme was evaluated by the pNPG method (p-nitrophenyl-*α*-D-glucopyranoside) with some modifications.* Miconia* sp. extract at different concentrations (1, 2, 3, 4, and 5 *μ*g/mL) was used as a vehicle a buffer of sodium phosphate mentioned above. Acarbose at different concentrations (62.5, 125, 250, 500, and 1000 *μ*g/mL) is positive control, using buffer sodium phosphate as a vehicle. *α*-Glucosidase enzyme (Sigma-Aldrich # G5003-100UN) at a concentration of 0.2 U/mL using phosphate buffer as a vehicle. Additionally, a preparation of pNPG 2 mM (Sigma-Aldrich # N1377-1G) was used as a substrate in the same vehicle. After that, in a 96-well microplate, 25 *μ*L of the substances was mixed with the enzyme to the assay: treatment 1 [*Miconia* sp. extracts], treatment 2 [phosphate buffer with 1% ethanol (negative control)], and treatment 3 [acarbose (positive control)]. Besides the above treatments, 25 *μ*L of the enzyme suspension was added and incubated at 37°C for 15 min; immediately after that, 50 *μ*L of pNPG was added and incubated at 37°C for 10 min; then, the reaction was stopped by adding 50 *μ*L sodium carbonate (0.2 M).

Finally, the optical density at 405 nm was measured. The absorbance rates were used to calculate the percent inhibition of each treatment using the following equation:

(2) $$\begin{array}{c} \%\;\;\text{Inhibition}=\frac{\left(\text{Abs control}-\text{Abs treatment}\right)}{\text{Abs control}}\times 100. \end{array}$$

In this equation, “Abs treatment” is the product's light absorption of the enzyme-substrate reaction in the presence of the* Miconia* sp., acarbose or negative control as appropriate. “Abs control” is the product's light absorption of the enzyme-substrate reaction in the presence of phosphate buffer as a treatment. Percent inhibitions using a statistical package (SPSS v.20) were used to determine the inhibitory concentration 50 (IC_50_) that is the amount of treatment necessary to inhibit the enzyme 50% [4, 13–15].

### 2.7. Cell Proliferation Assay

Cell proliferation with MTT colorimetric method (3-(4,5-dimethylthiazol-2-y1)-2,5-diphenyltetrazolium bromide) was evaluated. The VERO cell (monkey kidney epithelial) lines are widely used in research to evaluate the effect of chemicals and toxins in mammals [16]. 1 × 10^5^ cells/well were seeded in a 96-well microplate and adjusted to a final volume of 200 *μ*L with culture medium (DMEM supplemented with 10% FBS) and incubated at 37°C in an atmosphere of 5% CO_2_. After 24 h and until reaching a layer of 80% confluence, the culture medium was removed and cells were washed with phosphates (PBS), a buffered saline solution; further substances were added and evaluated: treatment 1 [extracts of* Miconia* sp. (62.5, 125, 250, 500, and 1000 *μ*g/mL)], treatment 2 [DMEM with 1% ethanol (negative control)], and treatment 3 [cells with culture medium (reference of proliferation)]. After that, the microplate was incubated for 24 h at the above conditions. Then, 10 *μ*L MTT (5 mg/mL) were added to each well and incubated again for 4 h; the culture medium was removed and cells were washed with PBS. Immediately afterwards, 200 *μ*L dimethyl sulfoxide (DMSO) was added; then the optical density was measured at 570 nm. By the following formula the percentage of cell proliferation was determined:

(3) $$\begin{array}{c} \%\;\;\text{cell proliferation}=\frac{\text{Abs treatment}}{\text{Abs control}}\times 100. \end{array}$$

In this equation, “Abs treatment” is the product's light absorption of the treated cells and “Abs control” is the product's light absorption of cells used as proliferation reference. The IC_50_ is calculated with the percentages of cell proliferation and with the support of SPSS v.20 [17, 18].

## 3. Results

In this study, different biological activities of ethanolic extract of* Miconia* sp. were evaluated in their effect on expression of mRNA PPAR*γ*, lipid accumulation during adipogenesis, the activity of *α*-amylase and *α*-glucosidase, and the effect on proliferation of VERO cell line.

**Figure 1:**
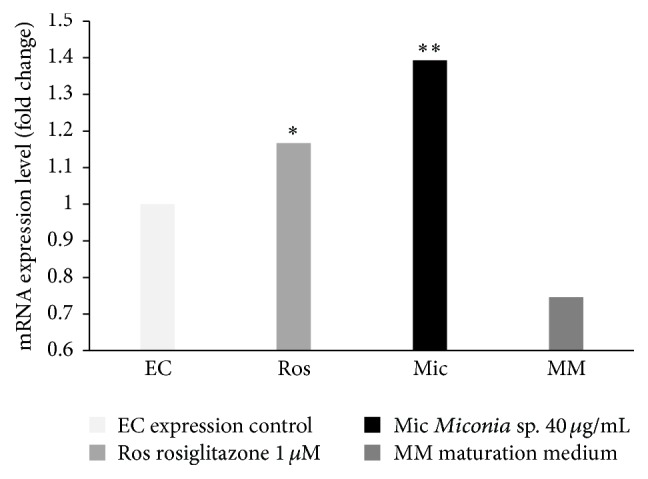
Effect of different compounds on the expression of mRNA PPAR*γ*. On day 10 the treatments were applied; Group 1: DMEM with 0.1% ethanol (EC), Group 2: MM and rosiglitazone (Ros) as a positive control, Group 3: MM and* Miconia* (Mic), and Group 4: MM. On day 11 of adipocyte differentiation the total RNA was isolated. ^*∗*^ *P* ≤ 0.05: there is a significant difference with the control of expression; ^*∗∗*^ *P* ≤ 0.05: there is a significant difference with the control of expression and rosiglitazone (*n* = 3).

In determining the expression level of mRNA of PPAR*γ* in mature mouse adipocytes, Group 1 was used as an expression control, which was normalized to a value of 1. Therefore, the values above 1 indicate an overexpression or upregulation. In Group 2 the value in the expression levels was 1.166 ± 0.007 fold change; at the same time, in Group 3 a value was 1.393 ± 0.008 and finally Group 4 showed a value of 0.746 ± 0.034 fold change (Figure 1).

*Miconia* sp. 40 *μ*g/mL produced upregulation in the expression of mRNA of PPAR*γ* with a value greater than that of the drug rosiglitazone, which increases the expression of PPAR*γ* as expected. The use of maturation medium with both extracts as rosiglitazone did not increase by itself the expression levels of the gene PPAR*γ*.

In the lipid accumulation test during adipogenesis with different treatments, Group 1 (Dm) showed absorbance of 0.137; this value represents the accumulated lipids during adipogenesis induced differentiation medium already established. At the same time, Group 2 (Dm +* Miconia* sp. 40 *μ*g/mL) showed absorbance of 0.184; Group 3 (Dm + rosiglitazone 1 *μ*M) showed an absorbance value of 0.177.

**Figure 2:**
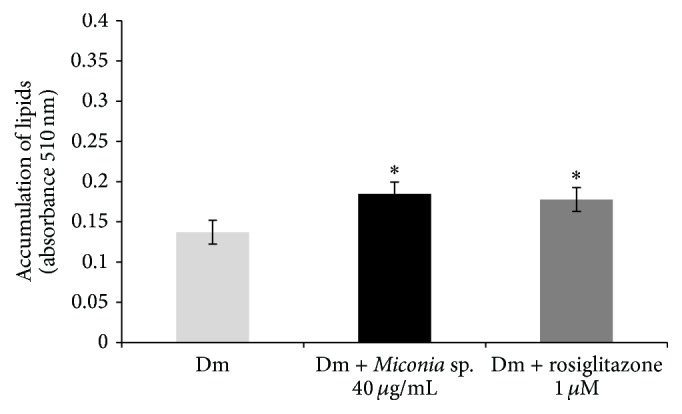
Effect of different substances in lipid accumulation during adipogenesis in 3T3-L1 cells. The treatments were applied at day 0 of differentiation and withdrawn at day 2. On day 10 when adipocytes reached maturity, the staining was performed. ^*∗*^ *P* ≤ 0.05 significant difference between treatments and Dm (*n* = 3).

Taking the absorbance value from Group 1 as 100% lipid accumulation, when extract* Miconia* sp. was added to the differentiation medium, lipid accumulation was increased to 34.57% compared to Group 1. Like the* Miconia* sp. extract, adding the drug rosiglitazone to differentiation medium, it causes an increase of 29.55% compared to Group 1. The increased absorbance of the extract and the drug is significant compared with Dm;* Miconia* sp. presented a very similar result to rosiglitazone, difference between them not of great significance (Figure 2).

In the *α*-amylase inhibition assay, different treatments to the mixture of enzyme-substrate reaction were added, obtaining the following results: for treatment 1,* Miconia* sp. presented IC_50_ of 28.23 ± 2.15 *μ*g/mL. At the same time, for treatment 2, phosphate buffer and 2% ethanol (negative control) showed no inhibition by the vehicle used; for treatment 3: acarbose presented IC_50_ of 993.84 ± 15.13 *μ*g/mL (positive control).

According to the results and despite being a complete extract, the extract of* Miconia* sp. reveals a greater capacity than acarbose to inhibit 50% of enzyme activity; this difference between the two treatments is statistically significant (Table 1).

In the inhibition assay of *α*-glucosidase, the following results were obtained: for treatment 1,* Miconia* sp. extract provided IC_50_ of 1.95 ± 0.15 *μ*g/mL. At the same time, for treatment 2, phosphate buffer with 1% ethanol (negative control) did not show any inhibitory effect on the enzyme; for treatment 3, acarbose (positive control) showed IC_50_ of 331.00 ± 72.08 *μ*g/mL (Table 1).

*Miconia* sp. extract also has a greater inhibition than acarbose, and acarbose is used as a reference compound in the inhibition of these enzymes. The differences in the treatments are statistically significant.

In determining the proliferation on the VERO cell line, IC_50_ of 314.54 ± 45.40 mg/mL considered toxic was obtained for* Miconia* sp. extract.

## 4. Discussion

PPAR*γ* is a nuclear receptor that acts as a transcription factor; it improves insulin sensitivity by the cells and enhances glucose utilization [19]. A diverse set of natural and synthetic molecules is classified as ligands and can induce activation and that expression of PPAR*γ*. These ligands include nutrients, endogenous ligands, and drugs [20]; one of those drugs is thiazolidinediones (TZDs), such as rosiglitazone.* Miconia* sp. showed an increased mRNA expression of PPAR*γ*, even more than rosiglitazone. Moreover,* Miconia* sp. was able to increase lipid accumulation during adipogenesis in 3T3 cell line L-1 similar to positive control rosiglitazone. It is known that PPAR*γ* is the master regulator of adipocyte differentiation, and during adipogenesis PPAR*γ* is induced [21]. PPAR*γ* activation in adipocytes ensures proper balance and secretion of adipokines, such as leptin and adiponectin; they are mediators in insulin action in peripheral tissues, which causes insulin sensitivity throughout the body [22]. The products mentioned above that stimulate the production of PPAR*γ* are candidates to induce the proper functioning of insulin and recognition, considering them as potential antidiabetic agents.

The results suggest that the presence of secondary metabolites could be involved in upregulation of PPAR*γ* gen. Quercetin, catechin, and kaempferol have been reported in some species of* Miconia* sp. and these compounds could act as ligands of PPAR*γ* [6, 21] just as rosiglitazone does. Some TZDs as rosiglitazone have been associated with a significant increase of cardiovascular diseases. For this reason, FDA restricted their prescription in the United States [23].

Avoiding the increase of postprandial glucose is important to keep the glycemic levels low in diabetic patients. The inhibition of *α*-amylase and *α*-glucosidase present in the gastrointestinal tract could keep the levels of glycemia low. Drugs inhibit these enzymes, such as acarbose, miglitol, voglibose, nojirimycin, and 1-deoxynojirimycin, which allow the slow absorption of carbohydrates [23]. The ethanolic extract of* Miconia* sp. inhibits *α*-amylase by 50% to a less concentration than the acarbose drug. Additionally, the *α*-glucosidase is inhibited by* Miconia* sp. at low concentrations, lower than the drug. Therefore, it is believed that in the presence of molecules with antihyperglycemic effect in* in vitro* model, these compounds could be an alternative to existing treatments or adjunctive to them, which may have undesired side effects.

*Miconia* sp. showed cytotoxicity at a greater concentration than necessary to increase expression of PPAR*γ*, increase lipid accumulation, and inhibit *α*-glucosidase and *α*-amylase. There are reports that the species of the genus* Miconia*,* M. stenostachya*,* M. cabucu*,* M. albicans*, and* M. rubiginosa,* lack cytotoxicity or mutagenicity at lower concentrations of 100 *μ*g/mL [6].

## 5. Conclusions

The ethanolic extract of* Miconia* sp. showed increase of the level of mRNA expression of PPAR*γ* at a significantly higher level than rosiglitazone (drug). Also,* Miconia* sp. showed inhibition of the enzymes *α*-glucosidase and *α*-amylase with IC_50_ lower than acarbose (drug) and furthermore increase the capacity of lipid accumulation during adipogenesis, similar to the drug rosiglitazone. At the same time,* Miconia* sp. showed cytotoxicity on VERO cells with a concentration higher than that presenting biological activity. For this reason, the compounds present in the ethanolic extract of* Miconia* sp. can be an alternative for the treatment of diabetes mellitus or like an adjunctive, with the recommendation of continuing with in vivo tests and elucidation of bioactive compounds.

## Figures and Tables

**Table 1:** Enzymatic inhibition from acarbose and *Miconia *sp. extract.

Treatment	*α*-Amylase (IC_50_ ± DE *μ*g/mL)	*α*-Glucosidase (IC_50_ ± DE *μ*g/mL)
*Miconia* ^*∗*^	28.23 ± 2.15	1.95 ± 0.15
Acarbose (positive control)	993.84 ± 157.13	331.00 ± 72.08
Phosphate buffer with 1% ethanol (negative control)	NI	NI

Data are expressed as mean ± the SD (*n* = 3). NI: no inhibition.  ^*∗*^ *P* ≤ 0.05 significant difference with acarbose.
